# Case Report: Buccal administration of hydrogen-producing blend after a mild traumatic brain injury in a professional athlete

**DOI:** 10.12688/f1000research.19739.1

**Published:** 2019-07-09

**Authors:** Dejan Javorac, Valdemar Stajer, Sergej M. Ostojic

**Affiliations:** 1 Faculty of Sport and Physical Education, University of Novi Sad, Novi Sad, 21000, Serbia; 2 Faculty of Health Sciences, University of Pecs, Pecs, H-7621, Hungary

**Keywords:** Traumatic brain injury, Concussion, Hydrogen, Recovery, Buccal administration, Athlete

## Abstract

**Background:** Sport-related mild traumatic brain injury (TBI) is a serious trauma that could impair brain function of an injured athlete. Treatment solutions for mild TBI typically concentrate on complete rest, while non-traditional therapeutic options remain largely ineffective. Molecular hydrogen (H _^2^_) is an innovative neuroprotective agent that can easily reach the brain, yet no data are available concerning its value as a first-aid intervention after a mild TBI.

**Case report:** This case report demonstrates the efficacy and safety of a hydrogen-producing dissolving tablet administered buccally during the first 24 hours post-injury in a professional soccer player who suffered a mild TBI. The patient received a formulated dosage of hydrogen every 2 hours, with the first intervention given immediately after an initial examination (~ 15 min after the injury).

The overall score for Sport Concussion Assessment Tool 2 (SCAT2), a standardized method of evaluating injured athletes for concussion, increased from 68 points (severe disruption) at baseline to 84 points (mild disruption) at 24-h follow-up. The patient reported no side effects of hydrogen intervention.

**Conclusions:** This case has demonstrated that intensive consecutive therapy with oral transmucosal hydrogen formulation is a beneficial strategy with regard to the reduction of presence and severity of symptoms of sport-related mild TBI.

## Introduction

Sport-related concussion or mild traumatic brain injury (TBI) is a perilous trauma that could impair the brain function of an injured athlete both acutely and in a long-term perspective. Up to 3.8 million sport-related concussions occur in the United States annually ^1^, with contact sports (e.g. football, wrestling, soccer, boxing, basketball, ice hockey) accounting for the greatest number of concussions ^2^. Common signs and symptoms of acute mild TBI include physical (e.g. headache, dizziness, nausea, vomiting, fatigue), cognitive (e.g. loss of consciousness, post-traumatic amnesia, difficulties with concentration and memory), emotional (e.g. irritability, depressed mood) and sleep-related problems (e.g. drowsiness, sleeping disturbances) ^3^. This happens probably due to a complex cascade of neurometabolic alterations in brain neurons damaged by head acceleration-deceleration mechanical forces ^4^, with mitochondrial dysfunction, energy metabolism disturbances, and excessive oxidative stress might play a role ^5^. Treatment solutions for sport-related concussion typically focus on complete rest at first (including a temporary withdrawal from practice and mental activities) followed by slow return-to-play activities monitored and clearance by a physician ^3^. Non-traditional therapies, such as dietary supplements, enteral nutrition, acupuncture, and music therapy, are being considered to combat mild TBI, yet the therapeutic options remain limited ^6^. Molecular hydrogen (H _2_) has recently been set forth as an innovative neuroprotective agent ^7^ that can easily reach hard-to-reach biocompartments ^8^, yet no data are available concerning its value as a first-aid intervention in mild TBI. This case report demonstrates the efficacy and safety of a hydrogen-producing blend administered buccally in a professional athlete that suffered a sport-related concussion.

## Case report

### Patient information

An apparently healthy young male professional soccer player (age 24 years, weight 75.1 kg, height 182.5 cm, professional experience 6 years) who suffered a sport-related concussion voluntarily participated in this case study. The injury occurred during a regular training session as a head-to-head collision with another player. There was a loss of consciousness for ~ 30 sec and the patient was immediately evaluated by a health care professional who confirmed the category of an injury by a physical examination. The patient had no history of concussion or other TBI in the past 6 months and no neurological, psychiatric or other chronic conditions. Written informed consent to be treated was obtained from the patient in accordance with the Declaration of Helsinki. Treatment using therapeutic hydrogen of athletes who experience a TBI was approved by the FSPE Applied Bioenergentics Lab (University of Novi Sad, Serbia; approval number, HBCS02-2019).

### Clinical findings

At the initial examination, the patient was profiled using Sport Concussion Assessment Tool 2 (SCAT2), a standardized method of evaluating injured athletes for concussion ^9^. The total number of concussion symptoms was 18 (out of maximal 22), with a symptom severity score of 53 (out of 132). Glasgow Coma Scale (GCS) score at baseline was 13 out of 15, corresponding to mild closed head injury. Total SCAT2 score was 68 out of 100 points, approximately 25.3% below normative values (91.08) ^10^. In addition, the patient has shown a diminished ability to keep balance during a single-leg stance test (SLST) for non-dominant foot (total number of errors 5 out of 10). 

### Therapeutic intervention

A hydrogen-producing blend was administered buccally during the first 24 hours post-injury. Hydrogen was used as an exclusive treatment (along with physical and mental rest) with the main aim to reduce symptoms and signs of concussion, and it was anticipated to accelerate the acute recovery. The patient received a formulated 700-mg tablet (producing ~ 80 mL of H _2_) every 2 hours throughout the monitoring period, with the first intervention given immediately after an initial examination (~ 15 min after the injury). The patient was requested to put a tablet into the mouth, preferentially between the gums and teeth, and keep it inside until full dissolution. A small quantity of tap water (~ 50 mL) has been provided for mouth rinsing to improve breaking up the tablet during each administration. Buccal administration was applied due to anticipated better bioavailability, more rapid onset of action, and decreased possible risk of vomiting, a well-known manifestation of concussion. The intervention was freely provided by HRW Natural Health Products Inc. (Drink HRW Rejuvenation, New Westminster, BC, Canada).

### Follow-up and outcomes

**Figure 1.**
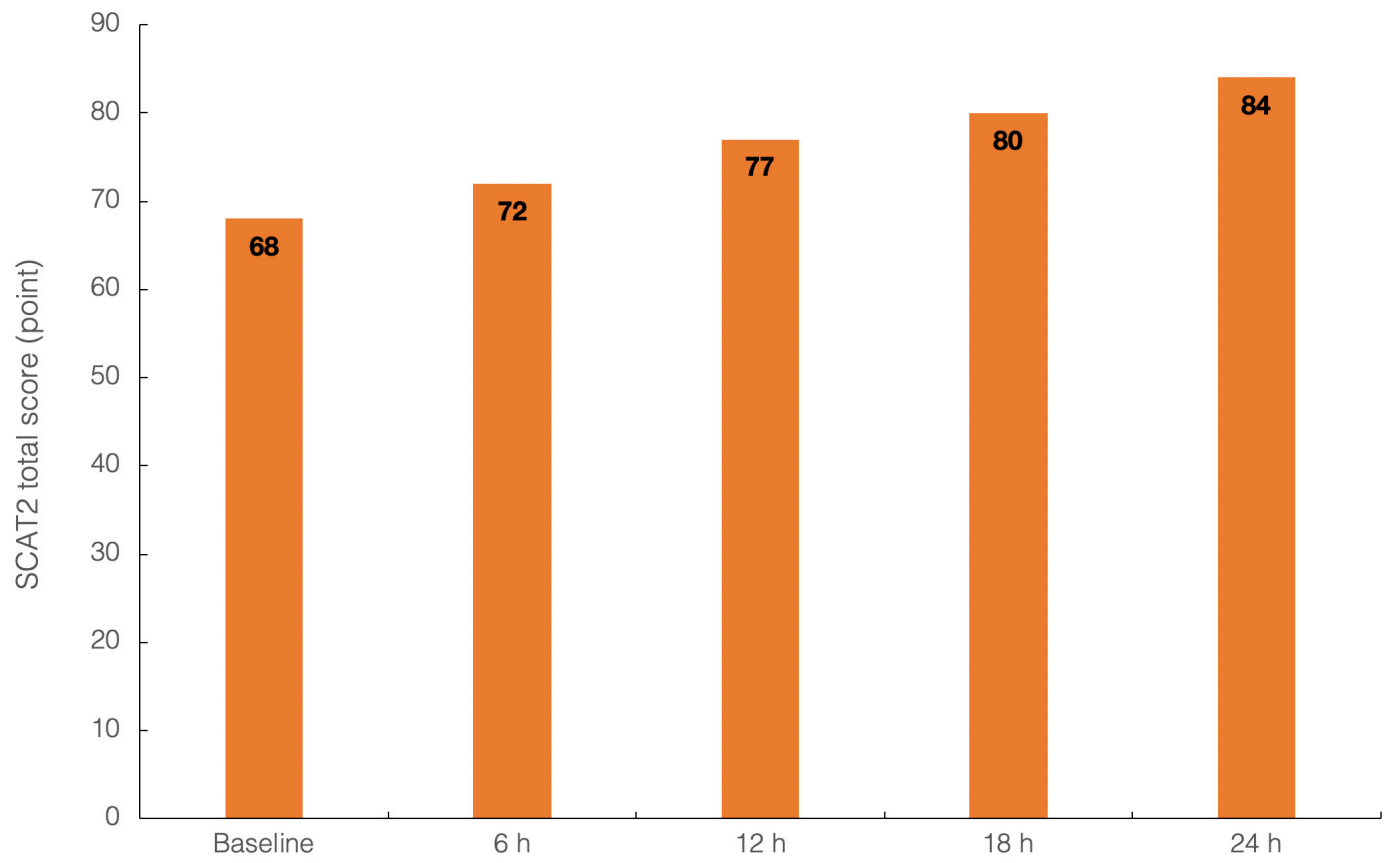
Sport Concussion Assessment Tool 2 (SCAT2) total scores at baseline and at each 6-h follow-up interval.

SCAT2 profiles were obtained at every 6 hours during the first 24 hours post-injury ( Figure 1). Total SCAT2 scores have risen from 68 (initial score) to 72 points at first re-evaluation period and continued to increase to 84 points at the final follow-up examination. In addition, the total number of concussion symptoms decreased to 9 at 24-h follow-up, with symptom severity score dropping to 12 (out of 132), while SLST balance test improved by 25% (total number of errors 2 out of 10 at 24-h follow-up). The patient reported no side effects of hydrogen intervention, as evaluated with an open-ended questionnaire administered at the end of each treatment period; the patient was asked if the intervention resulted in any adverse events, including oral irritation or discomfort, tingling in the mouth, dizziness, nausea or flushing. 

## Discussion

This case report implies the favorable effects of hydrogen-producing dissolving tablet administered buccally as a possible first-aid treatment to improve recovery in a professional athlete with a mild TBI. Previous animal studies have demonstrated neuroprotective effects of hydrogen against perinatal ^11^ and ischemic brain injury ^12^, or brain damage induced by neurosurgical intervention ^13^. In a recent pivotal study ^14^, TBI-challenged rats exhibited significant brain injuries that were characterized by decreased survival rate and increased blood-brain barrier permeability, brain edema, and neurological dysfunction, while hydrogen treatment ameliorated the consequences of TBI. The authors concluded that hydrogen could exert a neuroprotective effect against TBI and attenuate inflammation and/or oxidative stress ^14^, which put forward the intervention as a possible therapeutic strategy for TBI patients. Using an innovative route of administration and a benchmark tool (SCAT2) to evaluate injured athletes for concussion, we affirmed here that oral hydrogen ameliorates the presence and severity of symptoms of sport-related mild TBI. However, hydrogen has not been compared to placebo so the degree of advantage that hydrogen may provide to standard medical procedures for concussion (e.g. physical and mental rest) remains open to question. We strongly suggest further monitoring of the efficacy and safety of oral transmucosal hydrogen in sport-related mild TBIs on a larger similar case series using randomized controlled trials.

## Consent

Written informed consent was obtained from the patient for the publication of this case report, including any associated images.

## Data availability

All data underlying the results are available as part of the article. No additional source data are required.
