# Supplementary material for: Case Report: Buccal administration of hydrogen-producing blend after a mild traumatic brain injury in a professional athlete
Source: F1000Res. 2019 Jul 9;8:1024. [Version 1] doi: 10.12688/f1000research.19739.1 (PMC7308882; doi:10.12688/f1000research.19739.1)
Supplement: Peer review history

# Peer review: Reviewer response for version 1

**DOI:** 10.5256/f1000research.21648.r64103

**Published:** 2020-06-16

**Authors:** Katsuhiro Koyama, Shohei Dobashi

**Affiliations:** 1 Institute of Health and Sports Science & Medicine, Juntendo University, Inzai, Chiba, Japan; 2 Graduate School Department of Interdisciplinary Research, University of Yamanashi, Yamanashi, Japan

Sports-related mild traumatic brain injury (TBI) is one of social issues to solve in modern society, because the sports-related TBI increases subsequent mortality from neurodegenerative disorders, such as dementia. Given that if molecular hydrogen might easily reach injured brain area and induce some cytoprotective effects as the authors mentioned in this report, we evaluate the first-aid intervention by hydrogen administration as novel and innovative approach on the sports-related TBI.

 In order to potentiate the view that molecular hydrogen is beneficial for acute phase of sports-related TBI, we would like to propose the authors to consider some additional concerns as written below.

 Because of a case report, we understand that this study did not set placebo control trial (i.e., physical and mental rest without hydrogen treatment). However, we believe that the representation of the typical time course change in SCAT2 score without any specific medication will strongly enhance the therapeutic value of this intervention.

 Another point is that SCAT2 consists several clusters of assessment items (e.g., somatic, cognitive, emotional, and sleep-fatigue symptoms), nevertheless the authors displayed just only total SCAT2 score in this study. The information about which individual factor was improved by hydrogen treatment is so important to assess its efficacy and examine the target of the treatment. Thus, we would like to propose the authors to display the time course changes not only total score but also each score of SCAT2 symptoms.

Are enough details provided of any physical examination and diagnostic tests, treatment given and outcomes?

Yes

Is the case presented with sufficient detail to be useful for other practitioners?

Yes

Is sufficient discussion included of the importance of the findings and their relevance to future understanding of disease processes, diagnosis or treatment?

Yes

Is the background of the case’s history and progression described in sufficient detail?

Yes

Reviewer Expertise:

Exercise; Oxidative stress; Hypoxia; Molecular hydrogen;  Sport-related concussion;

We confirm that we have read this submission and believe that we have an appropriate level of expertise to confirm that it is of an acceptable scientific standard.

## References

[1] : Neurodegenerative Disease Mortality among Former Professional Soccer Players. *New England Journal of Medicine*.2019;381(19) : 10.1056/NEJMoa1908483 1801-1808 10.1056/NEJMoa1908483 PMC874703231633894

[2] : The Sport Concussion Assessment Tool (SCAT2) for evaluating civilian mild traumatic brain injury. A pilot normative study. *PLoS One*.2019;14(2) : 10.1371/journal.pone.0212541 e0212541 10.1371/journal.pone.0212541 30785950PMC6382274

# Peer review: Reviewer response for version 1

**DOI:** 10.5256/f1000research.21648.r56420

**Published:** 2019-11-29

**Authors:** Cheong Hwa Ooi

**Affiliations:** 1 Advanced Medical And Dental Institute, Universiti Sains Malaysia, Kepala Batas, Malaysia

Apart from physical and mental rest, there are limited options for clinical management of sport-related concussions or mild traumatic brain injury (TBI). This case report suggests that the ingestion of hydrogen-producing tablets may be a viable treatment to improve the recovery of the concussed footballer.

 The neuroprotective effects of molecular hydrogen against TBI  have been demonstrated in animal models, in the form of hydrogen gas inhalation or hydrogen-rich water administration. However, its efficacy in treating mild TBI in humans has not been examined. The buccal administration of the molecular hydrogen in the present case report represents an innovative immediate treatment approach to manage TBI in the field.

 The favorable outcomes of the intervention highlight the importance of a more extensive case series, and subsequently the need to conduct controlled trials in examining the therapeutic effects of molecular hydrogen on signs and symptoms of acute mild TBI in athletes.

Are enough details provided of any physical examination and diagnostic tests, treatment given and outcomes?

Yes

Is the case presented with sufficient detail to be useful for other practitioners?

Yes

Is sufficient discussion included of the importance of the findings and their relevance to future understanding of disease processes, diagnosis or treatment?

Yes

Is the background of the case’s history and progression described in sufficient detail?

Yes

Reviewer Expertise:

Heat Shock Proteins and Health; Eccentric Exercise-induced Muscle Damage; Molecular Hydrogen and Exercise Performance

I confirm that I have read this submission and believe that I have an appropriate level of expertise to confirm that it is of an acceptable scientific standard.
